# An Approach for the Generation of an Nth-Order Chaotic System with Hyperbolic Sine

**DOI:** 10.3390/e20040230

**Published:** 2018-03-27

**Authors:** Jizhao Liu, Jun Ma, Jing Lian, Pengbin Chang, Yide Ma

**Affiliations:** 1 School of Information Science and Engineering, Lanzhou University, Lanzhou 730000, China; 2 School of Physics and Electronic Information Engineering, Qinghai Normal University, Xining 810008, China

**Keywords:** chaos, chaotic circuit, ordinary differential equations, hyperbolic sine

## Abstract

Chaotic systems with hyperbolic sine nonlinearity have attracted the attention of researchers in the last two years. This paper introduces a new approach for generating a class of simple chaotic systems with hyperbolic sine. With nth-order ordinary differential equations (ODEs), any desirable order of chaotic systems with hyperbolic sine nonlinearity can be easily constructed. Fourth-order, fifth-order, and tenth-order chaotic systems are taken as examples to verify the theory. To achieve simplicity of the electrical circuit, two back-to-back diodes represent hyperbolic sine nonlinearity without any multiplier or subcircuits. Thus, these systems can achieve both physical simplicity and analytic complexity at the same time.

## 1. Introduction

Chaos is commonly associated with entropy [1]. For example, positive entropy is one of the most important ways to understand chaos [2]. It has been shown that positive entropy implies Li-Yorke chaos [3]. In the past three decades, a variety of chaotic systems have been proposed [4,5,6,7,8,9,10,11]. Some of them are generated by discrete chaotic maps such as the Logistics map [12], the tent map [13], the Hénon map [14], etc. The others are continuous-time chaotic systems [15]. These systems are in the form of autonomous ordinary differential equations (ODEs) with at least three variables and one nonlinearity [16]. This is because the Poincaré–Bendixson theorem implies that a two-dimensional continuous dynamical system cannot give rise to a chaotic system [17]. Today, continuous-time chaotic systems have many practical applications, to name just a few, they have been widely used in image encryption [18], secure communication [19], and liquid mixing [20]. These experiments have achieved good results. For example, Zhang and Chen have implemented a liquid mixer by using Chua’s circuit. Comparable experiments have shown that the mixing time in the sucrose dissolving processes can be changed dramatically along with various impeller/tank velocities.The chaotic perturbations have been verified to be an excellent candidate for improving the mixing efficiency [21]. Therefore, it is a significant task to design, analyze, and implement new continuous-time chaotic systems.

According to the research, a nonlinear term is very important for generating chaos. It can be a piecewise nonlinear function [22], a trigonometric function [23], an absolute value function [24], or a power function [25]. However, few of them can be hyperbolic sine functions [26]. This is because the graph of y=sinh(x) is upward-sloping, and increases faster as *x* increases. In 2011, Sprott and Munmuangsaen proposed an exponential chaotic system, which happens to be an example of the simplest chaotic system [27]. In the same year, Sprott used common resistors, capacitors, operational amplifiers, and a diode to successfully implement this system in a circuit [28]. Compared with chaotic systems with exponential nonlinearity, the chaotic system with hyperbolic sine nonlinearity has richer dynamic behavior because it is symmetrical and can exhibit symmetry breaking, and offers the possibility that attractors will split or merge as some bifurcation parameter is changed. In the last two years, these systems have attracted the attention of researchers.

Piper and Sprott proposed three kinds of simplicity in 2010, namely: mathematical simplicity, circuit simplicity, and simplicity from a practical standpoint [29]. From this perspective, many chaotic systems such as non-autonomous circuits are not “simple” because they achieve physical simplicity at the expense of analytic complexity, or vice versa [30]. Therefore, finding a simple chaotic system is a challenging task.

In this work, a class of simple chaotic systems with hyperbolic sine nonlinearity is proposed. With general nth-order ordinary differential equations (ODEs), any desirable order of hyperbolic sine chaotic systems could be constructed. Fourth-order, fifth-order, and tenth-order chaotic systems are taken as examples. To achieve simplicity of an electrical circuit, two back-to-back diodes represent hyperbolic sine nonlinearity without any multiplier or subcircuits. Thus, these systems could satisfy all three kinds of simplicities at the same time.

The rest of the paper is organized as follows. The general equations for generating chaos with hyperbolic sine nonlinearity are given in Section 2. Then, fourth-order, fifth-order, and tenth-order chaotic systems with hyperbolic sine nonlinearity are discussed in Section 3, Section 4 and Section 5. Some problems and future work are discussed in Section 6. Conclusions are drawn in Section 7.

## 2. General Chaotic System with Hyperbolic Sine

In this section, a general chaotic system with hyperbolic sine will be proposed.

We begin with a jerk system, because it can offer exceptionally simple notation for higher-order systems. The general jerk equation is described by

(1) $$\dddot{x}+\alpha \ddot{x}+\beta f\left(\dot{x}\right)+x=0$$

where α and β are real parameters, $f(\dot{x})$ is a nonlinear function, $\dot{x}=\frac{dx}{dt}$ is called velocity, $\ddot{x}=\frac{d^{2}x}{dt^{2}}$ is called acceleration, $\dddot{x}=\frac{d^{3}x}{dt^{3}}$ is called jerk. In order to more easily design a high-order chaotic system. Equation (1) is rewritten by replacing *x* with $x_{1}$, the velocity of *x* with $x_{2}$, and the acceleration of *x* with $x_{3}$. The general jerk equation can be written by

(2) $$\left\{\begin{array}{cc} \dot{x_{1}} & =x_{2}\\ \dot{x_{2}} & =x_{3}\\ \dot{x_{3}} & =-\alpha x_{3}-\beta f\left(x_{2}\right)-x_{1} \end{array}\right.$$

The last equation is called the jerk equation. In this dynamic system, the nonlinearity is $\beta f(x_{2})$. The common forms of $f(x_{2})$ are quadratic function, absolute value function, and piecewise-linear function. In recent years, a simple chaotic system with hyperbolic sine nonlinearity has been proposed [31,32]. The equations are given by

(3) $$\dddot{x}+0.75\ddot{x}+x+\rho \sin h\left(\psi \dot{x}\right)=0$$

where $\sin h\left(\psi \dot{x}\right)=\frac{e^{\psi \dot{x}}-e^{-\psi \dot{x}}}{2}$, $\rho =1.2\times 10^{-6}$ and $\psi =\frac{1}{0.026}$, which have been chosen to facilitate circuit implementation using diodes.

Based on these equations, a general high-order chaotic system for n>3 can be constructed, where *n* is the order of the system; it is described by

(4) $$\left\{\begin{array}{c} \dot{x_{1}}=x_{2}-x_{1}\\ \dot{x_{2}}=x_{3}-x_{2}\\ \cdots \\ \dot{x_{n-3}}=x_{n-2}-x_{n-3}\\ \dot{x_{n-2}}=x_{n-1}\\ \dot{x_{n-1}}=x_{n}\\ \dot{x_{n}}=-x_{n}-f\left(x_{n-1}\right)-nx_{n-2}-nx_{n-3}-\cdots -\frac{1}{2n}x_{1} \end{array}\right.$$

In our system, the nonlinear function is $f(x_{n-1})$,which is defined by $f\left(x_{n-1}\right)=\rho \sin h\left(\psi x_{n-1}\right)$. In these systems, the nonlinearity can be conveniently approximated using back-to-back diodes without any other components or subcircuits.

## 3. Fourth-Order Case

### 3.1. Numerical Solution of Fourth-Order Chaotic System with Hyperbolic Sine

According to the Equations in (4), the fourth-order chaotic system with hyperbolic sine is described by

(5) $$\left\{\begin{array}{c} \dot{x_{1}}=x_{2}-x_{1}\\ \dot{x_{2}}=x_{3}\\ \dot{x_{3}}=x_{4}\\ \dot{x_{4}}=-x_{4}-1.2\times 10^{-6}\sin h\left(x_{3}/0.026\right)-4x_{2}-0.125x_{1} \end{array}\right.$$

**Figure 1:**
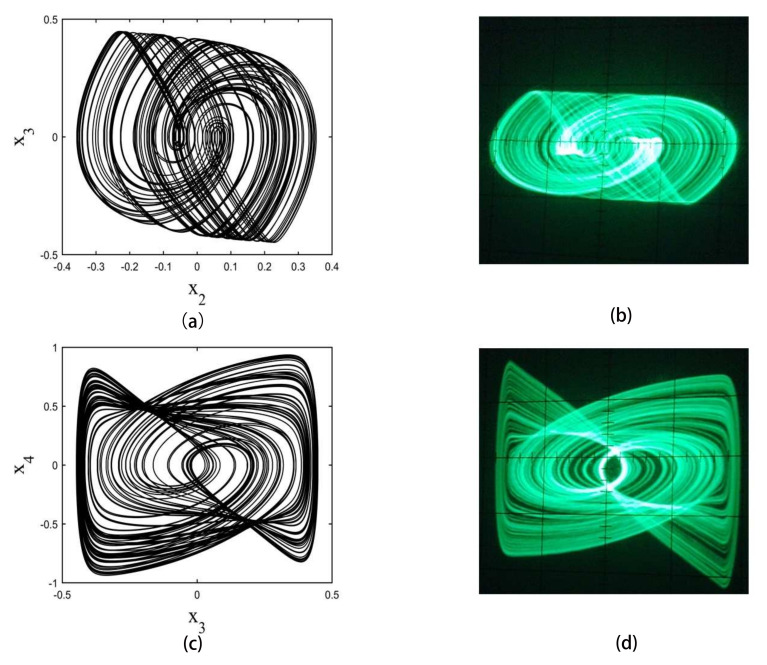
Phase space plots of numerical calculation and circuit implementation results for a fourth-order hyperbolic sine chaotic system. (**a**,**c**) are numerical calculations of the phase space plot of x${}_{2}$–x${}_{3}$ plane and x${}_{3}$–x${}_{4}$ plane, respectively; (**b**,**d**) are circuit implementation results of phase space plot of x${}_{2}$–x${}_{3}$ plane and x${}_{3}$–x${}_{4}$ plane, respectively.

The strange attractors are shown in Figure 1. The calculation was performed using a fourth-order Runge–Kutta integrator with a step size of 0.001. Initial conditions are not critical. In these experiments, the initial condition is set to be $\left(x_{1},x_{2},x_{3},x_{4}\right)=\left(0.1,0.1,0.1,0.1\right)$

Lyapunov exponents characterize the average exponential rate of separation of infinitesimally close trajectories in state space as time tends to infinity.

Consider the following n-dimensional dynamic system: $\dot{X}=F\left(X\right)$, where $X={\left(x_{1},x_{2},\cdots ,x_{n}\right)}^{T}\in R^{n}$, $F\left(X\right)={\left(f_{1}\left(X\right),f_{2}\left(X\right),\cdots ,f_{n}\left(x\right)\right)}^{T}$, $\dot{X}=\frac{dX}{dt}$. The two trajectories in phase space with initial separation $\delta X_{0}$ diverge (provided that the divergence can be treated within the linearized approximation) at a rate given by $\left|\delta X\left(t\right)\right|\approx e^{\lambda t}\left|\delta X_{0}\right|$, where λ is the Lyapunov exponent.

The complete procedure to evaluate the Lyapunov exponents is as follows [33]:

1. Start with any initial condition in the basin of attraction.
2. Iterate until the orbit is on the attractor.
3. Set Lyapunov exponent sphere, the initial center of the sphere is $X0={\left(x_{10},x_{20},\cdots ,x_{n0}\right)}^{T}$, which is on the system orbit. Select nearby points which is separated by d0. The coordinate of these points are
  (6) $$\begin{array}{c} X1={\left(x_{11},x_{21},\cdots ,x_{n1}\right)}^{T}={\left(x_{10}+d0,x_{20},\cdots ,x_{n0}\right)}^{T}\\ X2={\left(x_{12},x_{22},\cdots ,x_{n2}\right)}^{T}={\left(x_{10},x_{20}+d0,\cdots ,x_{n0}\right)}^{T}\\ \cdots \\ Xn={\left(x_{1n},x_{2n},\cdots ,x_{nn}\right)}^{T}={\left(x_{10},x_{20},\cdots ,x_{n0}+d0\right)}^{T} \end{array}$$
  In this calculation, the value of d0 is set to be $10^{-8}$.
4. Advance all orbits one iteration and calculate the new separation. The new points are
  (7) $$\begin{array}{c} X0^{\prime }={\left(x_{10}^{\prime },x_{20}^{\prime },\cdots ,x_{n0}^{\prime }\right)}^{T}\\ X1^{\prime }={\left(x_{11}^{\prime },x_{21}^{\prime },\cdots ,x_{n1}^{\prime }\right)}^{T}\\ X2^{\prime }={\left(x_{12}^{\prime },x_{22}^{\prime },\cdots ,x_{n2}^{\prime }\right)}^{T}\\ \cdots \\ Xn^{\prime }={\left(x_{1n}^{\prime },x_{2n}^{\prime },\cdots ,x_{nn}^{\prime }\right)}^{T} \end{array}$$
  The separation is calculated from the sum of the squares of the differences in each variable. For this n-dimensional system, set
  (8) $$\begin{array}{c} e1=X1^{\prime }-X0^{\prime }=\left(x_{11}^{\prime }-x_{10}^{\prime },x_{21}^{\prime }-x_{20}^{\prime },\cdots ,x_{n1}^{\prime }-x_{n0}^{\prime }\right)\\ e2=X2^{\prime }-X0^{\prime }=\left(x_{12}^{\prime }-x_{10}^{\prime },x_{22}^{\prime }-x_{20}^{\prime },\cdots ,x_{n2}^{\prime }-x_{n0}^{\prime }\right)\\ \cdots \\ en=Xn^{\prime }-X0^{\prime }=\left(x_{1n}^{\prime }-x_{10}^{\prime },x_{2n}^{\prime }-x_{20}^{\prime },\cdots ,x_{nn}^{\prime }-x_{n0}^{\prime }\right) \end{array}$$
  therefore, the new separation is d1=||e1||,d2=||e2||,⋯,dn=||en||
5. Evaluate the logarithm. log|d1/d0|,log|d2/d0|,⋯,log|dn/d0|.
6. Readjust e1,e2,⋯,en by using Gram–Schmidt process.
7. Readjust the orbits so their separation are in the same direction as original one.
  (9) $$\begin{array}{c} X0^{\prime \prime }={\left(x_{10}^{\prime },x_{20}^{\prime },\cdots ,x_{n0}^{\prime }\right)}^{T}\\ X1^{\prime \prime }={\left(x_{10}^{\prime }+e1\left(1\right)\times d1/d0,x_{20}^{\prime }+e1\left(2\right)\times d1/d0,\cdots ,x_{n0}^{\prime }+e1\left(n\right)\times d1/d0\right)}^{T}\\ X2^{\prime \prime }={\left(x_{10}^{\prime }+e2\left(1\right)\times d2/d0,x_{20}^{\prime }+e2\left(2\right)\times d2/d0,\cdots ,x_{n0}^{\prime }+e2\left(n\right)\times d2/d0\right)}^{T}\\ \cdots \\ Xn^{\prime \prime }={\left(x_{10}^{\prime }+en\left(1\right)\times dn/d0,x_{20}^{\prime }+en\left(2\right)\times dn/d0,\cdots ,x_{n0}^{\prime }+en\left(n\right)\times dn/d0\right)}^{T} \end{array}$$
8. Repeat step 4–6 and calculate the average of step 5.

Based on the above algorithm, the Lyapunov exponents are calculated to be (0.3008,0,−1.004,−1.2968).

To study the dynamical behavior further, a coefficient 1 is replaced by a control parameter *A* which is varied over the range A∈[0,2]. The equations are given by

(10) $$\left\{\begin{array}{c} \dot{x_{1}}=x_{2}-x_{1}\\ \dot{x_{2}}=x_{3}\\ \dot{x_{3}}=x_{4}\\ \dot{x_{4}}=-Ax_{4}-1.2\times 10^{-6}\sin h\left(x_{3}/0.26\right)-4x_{2}-0.125x_{1} \end{array}\right.$$

**Figure 2:**
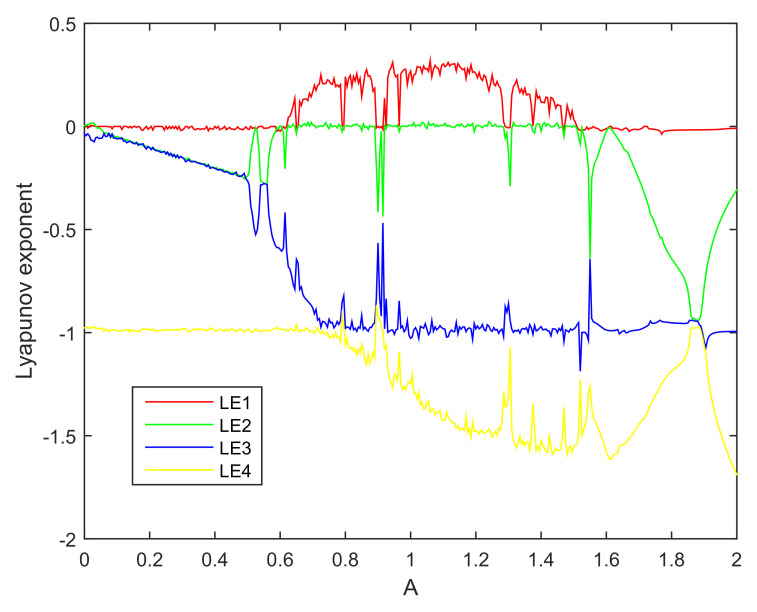
Lyapunov exponent spectrum of a fourth-order chaotic system.

A Lyapunov exponent spectrum is plotted in Figure 2.

In Figure 2, periodic dynamics correspond to the largest Lyapunov exponent (LLE) that is equal to zero. Chaotic behavior corresponds to the LLE that is greater than zero. For A∈[0.61,1.52], except of small windows in which the LLE is equal to zero, the largest Lyapunov exponents is positive, thereby confirming that the system is chaotic. For A<0.61 and A>1.52 and some aforementioned small windows inside the chaotic region, the LLE is equal to zero, the system in these regions exhibits limit cycle.

### 3.2. Circuit Implementation of Fourth-Order Chaotic System with Hyperbolic Sine

**Figure 3:**
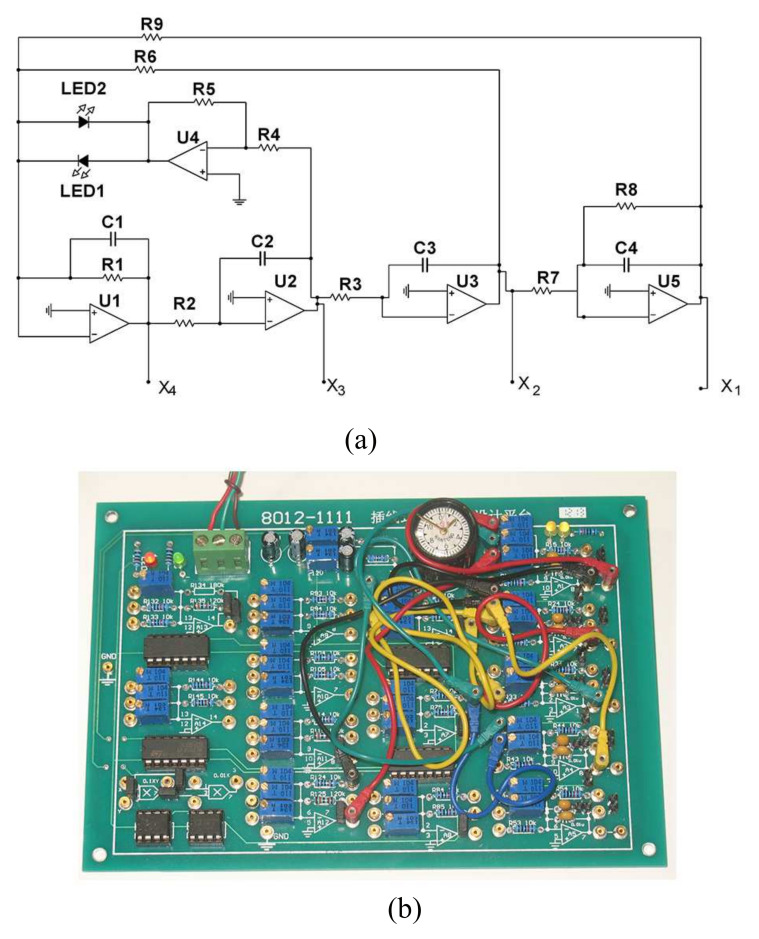
Circuit scheme and physical board of fourth-order chaotic system with hyperbolic sine. (**a**) is the circuit schematic of the fourth-order chaotic system; (**b**) is the physical board of the fourth-order chaotic system.

The scheme and physical board of a fourth-order system are shown in Figure 3.

In this circuit, U1, U2, U3 and U5 are configured as integrators. U4 is configured as voltage follower. The Shockley diode equation is $I=I_{S}\left(e^{\frac{V_{D}}{nV_{T}}}-1\right)$, where $I_{S}$ is the saturation current or scale current of the diode, $V_{T}$ is the thermal voltage (kT/q, about 26 mV at normal temperatures), and *n* is known as the diode ideality factor (for silicon diodes, *n* is approximately 1 to 2). Therefore, the relationship between the *I*–*V* characteristic and the two back-to-back diodes is $I=I_{LED1}-I_{LED2}=I_{S}\left(e^{\frac{V_{D}}{nV_{T}}}-1\right)-I_{s}\left(e^{\frac{-V_{D}}{nV_{T}}}-1\right)=I_{S}\left(e^{\frac{V_{D}}{nV_{T}}}-e^{\frac{-V_{D}}{nV_{T}}}\right)=2I_{S}\sin h\left(\frac{V_{D}}{nV_{T}}\right)$. Thus, by applying the Kirchhoff’s laws into the circuit of Figure 3, its mathematical model, given by the system of four differential equations, is obtained as:

(11) $$\left\{\begin{array}{c} C_{4}\frac{dV_{C_{4}}}{dt}+\frac{V_{C_{4}}}{R_{8}}+\frac{V_{C_{3}}}{R_{7}}=0\\ C_{3}\frac{dV_{C_{3}}}{dt}+\frac{V_{C_{2}}}{R_{3}}=0\\ C_{2}\frac{dV_{C_{2}}}{dt}+\frac{V_{C_{1}}}{R_{2}}=0\\ C_{1}\frac{dV_{C_{1}}}{dt}+\frac{V_{C_{1}}}{R_{1}}+\frac{R_{5}}{R_{4}}2I_{s}\sin h\left(\frac{V_{C_{2}}}{nV_{T}}\right)+\frac{V_{C_{3}}}{R_{6}}+\frac{V_{C_{4}}}{R_{9}}=0 \end{array}\right.$$

where $V_{C_{1}}$, $V_{C_{2}}$, $V_{C_{3}}$ and $V_{C_{4}}$ are voltages across the four capacitors C1, C2, C3 and C4, respectively. It can be rescaled by using dimensionless variables and parameters given by: $x_{1}=\frac{V_{C_{4}}}{nV_{T}}$, $x_{2}=\frac{V_{C_{3}}}{nV_{T}}$, $x_{3}=\frac{V_{C_{2}}}{nV_{T}}$, $x_{4}=\frac{V_{C_{1}}}{nV_{T}}$, which is Equation (10)

In this circuit, all resistors are set to be 10 kΩ with 10% tolerance, except R6=2.4 kΩ and R9=82 kΩ. All capacitors are set to be 0.01 μF with 10% tolerance. The operational amplifiers are TL084, and diodes are light emitting diodes.

The circuit offers excellent agreement with the numerical solution of the phase space plot, as shown in Figure 1.

## 4. Fifth-Order Case

### 4.1. Numerical Solution of Fifth-Order Chaotic System with Hyperbolic Sine

According to the Equations in (4), the fifth-order chaotic system with hyperbolic sine is described by

(12) $$\left\{\begin{array}{c} \dot{x_{1}}=x_{2}-x_{1}\\ \dot{x_{2}}=x_{3}-x_{2}\\ \dot{x_{3}}=x_{4}\\ \dot{x_{4}}=x_{5}\\ \dot{x_{5}}=-x_{5}-1.2\times 10^{-6}\sin h\left(x_{4}/0.026\right)-5x_{3}-5x_{2}-0.1x_{1} \end{array}\right.$$

**Figure 4:**
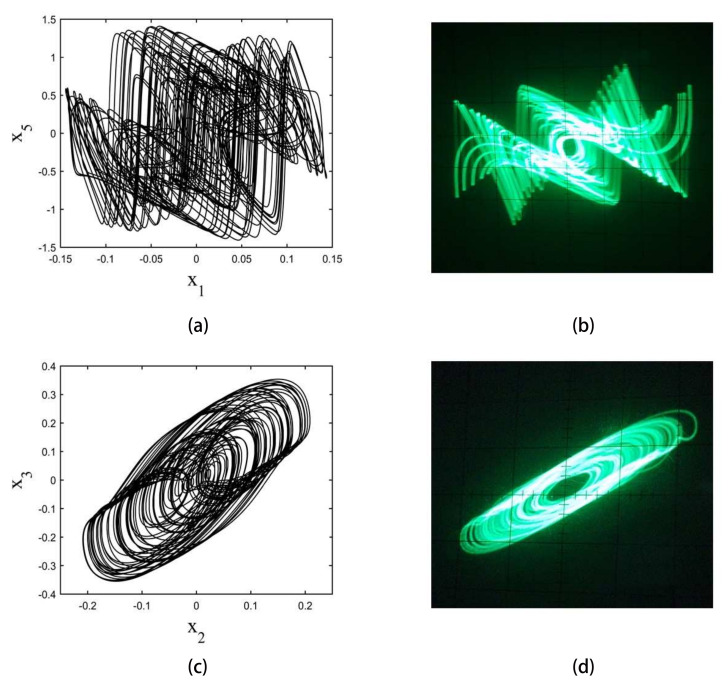
Phase space plots of numerical calculation and circuit implementation results for a fifth-order hyperbolic sine chaotic system. (**a**,**c**) are numerical calculations of the phase space plot of x${}_{1}$–x${}_{5}$ plane and x${}_{2}$–x${}_{3}$ plane, respectively; (**b**,**d**) are circuit implementation results of the phase space plot of a x${}_{1}$–x${}_{5}$ plane and x${}_{2}$–x${}_{3}$ plane, respectively.

The strange attractors are shown in Figure 4. The calculation was performed using a fourth-order Runge–Kutta integrator with a step size of 0.001. In these experiments, it is set to be $\left(x_{1},x_{2},x_{3},x_{4},x_{5}\right)=\left(0.1,0.1,0.1,0.1,0.1\right)$

Based on the algorithm in Section 3. The Lyapunov exponents are calculated to be (0.4591,0,−1.0186,−1.0877,−1.3528).

To study the dynamical behavior further, a coefficient 1 is replaced by a control parameter *A* which is varied over the range A∈[0,2]. The equations are given by

(13) $$\left\{\begin{array}{ccc} \dot{x_{1}} & =x_{2}-x_{1}\\ \dot{x_{2}} & =x_{3}-x_{2}\\ \dot{x_{3}} & =x_{4}\\ \dot{x_{4}} & =x_{5}\\ \dot{x_{5}} & =-Ax_{5}-1.2\times 10^{-6}\sin h\left(x_{4}/0.026\right)-5x_{3}-5x_{2} & -0.1x_{1} \end{array}\right.$$

**Figure 5:**
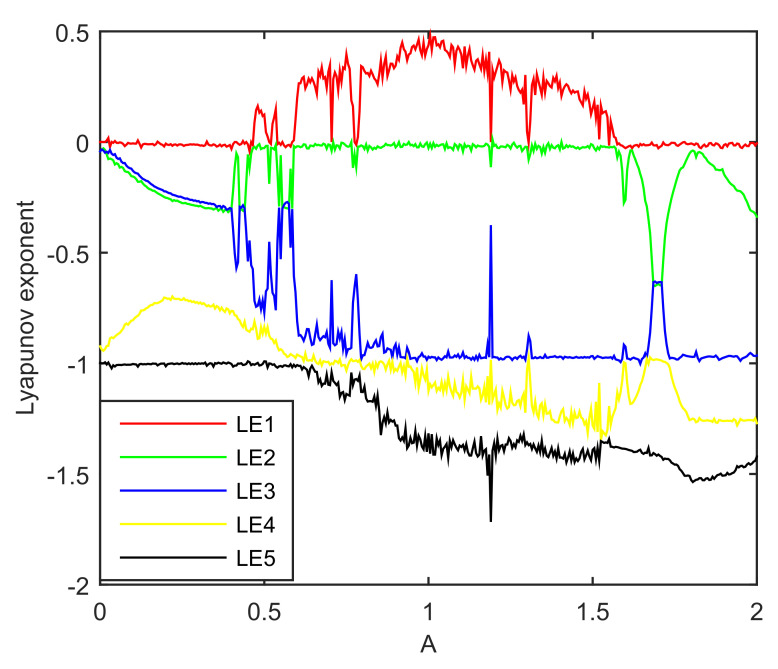
Lyapunov exponent spectrum of a fifth-order chaotic system.

The Lyapunov exponent spectrum is shown in Figure 5.

From Figure 5, For A∈[0.46,1.60], except of small windows in which the LLE is equal to zero, the largest Lyapunov exponents is positive, thereby confirming that the system is chaotic. For A<0.46 and A>1.60 and some aforementioned small windows inside the chaotic region, the LLE is equal to zero, the system in these regions exhibits limit cycle.

### 4.2. Circuit Implementation of Fifth-Order Chaotic System with Hyperbolic Sine

**Figure 6:**
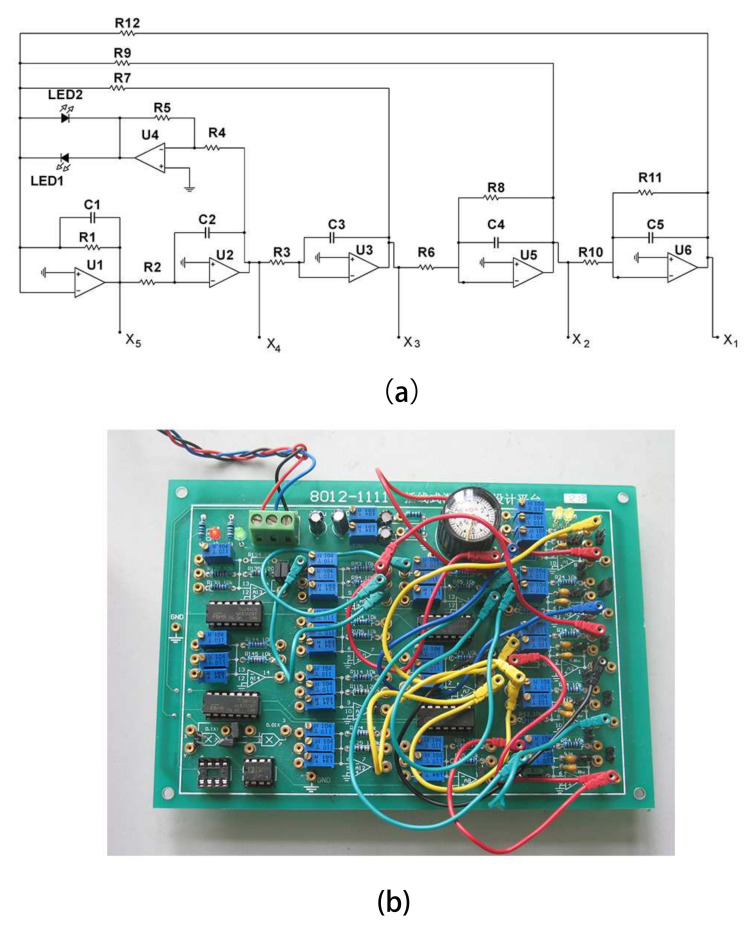
Circuit scheme and physical board of a fifth-order chaotic system with hyperbolic sine. (**a**) is the circuit schematic of the fifth-order chaotic system; (**b**) is the physical board of a fifth-order chaotic system.

The corresponding circuit of a fifth-order chaotic system with hyperbolic sine is shown in Figure 6.

With the analysis of Section 3, the mathematical model of this circuit is obtained as:

(14) $$\left\{\begin{array}{c} C_{5}\frac{dV_{C_{5}}}{dt}+\frac{V_{C_{5}}}{R_{11}}+\frac{V_{C_{4}}}{R_{10}}=0\\ C_{4}\frac{dV_{C_{4}}}{dt}+\frac{V_{C_{4}}}{R_{8}}+\frac{V_{C_{3}}}{R_{6}}=0\\ C_{3}\frac{dV_{C_{3}}}{dt}+\frac{V_{C_{2}}}{R_{3}}=0\\ C_{2}\frac{dV_{C_{2}}}{dt}+\frac{V_{C_{1}}}{R_{2}}=0\\ C_{1}\frac{dV_{C_{1}}}{dt}+\frac{V_{C_{1}}}{R_{1}}+\frac{R_{5}}{R_{4}}2I_{s}\sin h\left(\frac{V_{C_{2}}}{nV_{T}}\right)+\frac{V_{C_{3}}}{R_{7}}+\frac{V_{C_{4}}}{R_{9}}+\frac{V_{C_{5}}}{R_{12}}=0 \end{array}\right.$$

In this circuit, all capacitors are taken as 0.01 μF with 10% tolerance. All resistors are taken as 10 KΩ with 10% tolerance, except R7=2 KΩ, R9=100 KΩ, and R11=100 KΩ. The operational amplifiers are TL084 and the diodes are light emitting diodes.

The phase space plot of the circuit is shown in Figure 4.

## 5. Tenth-Order Case

According to the Equations in (4), the tenth-order chaotic system with hyperbolic sine is described by

(15) $$\left\{\begin{array}{cc} \dot{x_{1}}= & \;x_{2}-x_{1}\\ \dot{x_{2}}= & \;x_{3}-x_{2}\\ \dot{x_{3}}= & \;x_{4}-x_{3}\\ \dot{x_{4}}= & \;x_{5}-x_{4}\\ \dot{x_{5}}= & \;x_{6}-x_{5}\\ \dot{x_{6}}= & \;x_{7}-x_{6}\\ \dot{x_{7}}= & \;x_{8}-x_{7}\\ \dot{x_{8}}= & \;x_{9}\\ \dot{x_{9}}= & \;x_{10}\\ \dot{x_{10}}= & \;-x_{10}-1.2\times 10^{-6}\sin h\left(x_{9}/0.026\right)-10x_{8}-10x_{7}-10x_{6}\\ & \;-10x_{5}-10x_{4}-10x_{3}-10x_{2}-0.05x_{1} \end{array}\right.$$

**Figure 7:**
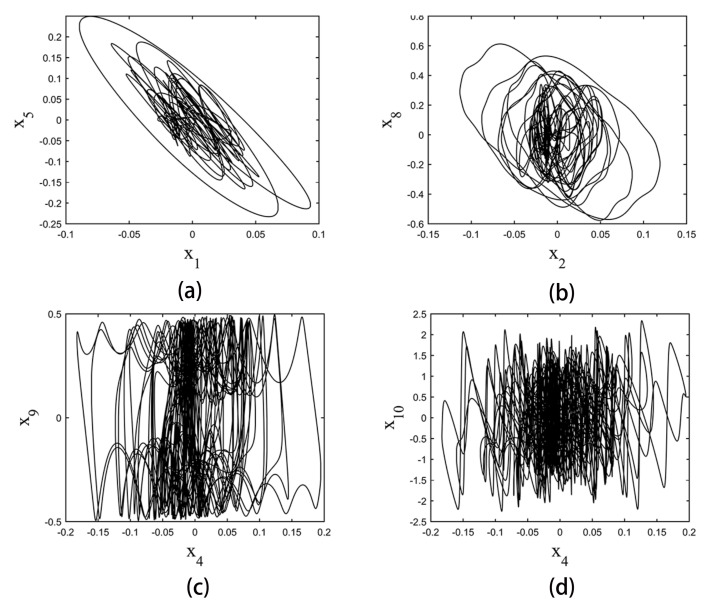
Some phase space plots of tenth-order hyperbolic sine chaotic system. (**a**) is the phase space plot of x${}_{1}$–x${}_{5}$ plane; (**b**) is the phase space plot of x${}_{2}$–x${}_{8}$ plane; (**c**) is the phase space plot of x${}_{4}$–x${}_{9}$ plane; (**d**) is the phase space plot of x${}_{4}$–x${}_{10}$ plane.

Some phase space plots of the strange attractors are shown in Figure 7. The calculation was performed using a fourth-order Runge–Kutta integrator with a step size of 0.001. Initial conditions are not critical. In these experiments, they are set to be $\left(x_{1},\;x_{2},\;x_{3},\;x_{4},\;x_{5},\;x_{6},\;x_{7},\;x_{8},\;x_{9},\;x_{10}\right)=\left(0.1,\;0.1,\;0.1,\;0.1,\;0.1,\;0.1,\;0.1,\;0.1,\;0.1,\;0.1\right)$.

Based on the algorithm in Section 3. The Lyapunov exponents are calculated to be (0.5306,0,−0.3990,−0.4735,−0.8917,−0.9969,−1.1466,−1.3829,−1.5461,−1.6939).

To study the dynamical behavior further, a coefficient 1 is replaced by a control parameter *A* which is varied over the range A∈[0,2]. The equations are given by

(16) $$\left\{\begin{array}{cc} \dot{x_{1}}= & \;x_{2}-x_{1}\\ \dot{x_{2}}= & \;x_{3}-x_{2}\\ \dot{x_{3}}= & \;x_{4}-x_{3}\\ \dot{x_{4}}= & \;x_{5}-x_{4}\\ \dot{x_{5}}= & \;x_{6}-x_{5}\\ \dot{x_{6}}= & \;x_{7}-x_{6}\\ \dot{x_{7}}= & \;x_{8}-x_{7}\\ \dot{x_{8}}= & \;x_{9}\\ \dot{x_{9}}= & \;x_{10}\\ \dot{x_{10}}= & \;-Ax_{10}-1.2\times 10^{-6}\sin h\left(x_{9}/0.026\right)-10x_{8}-10x_{7}\\ & \;-10x_{6}-10x_{5}-10x_{4}-10x_{3}-10x_{2}-0.05x_{1} \end{array}\right.$$

**Figure 8:**
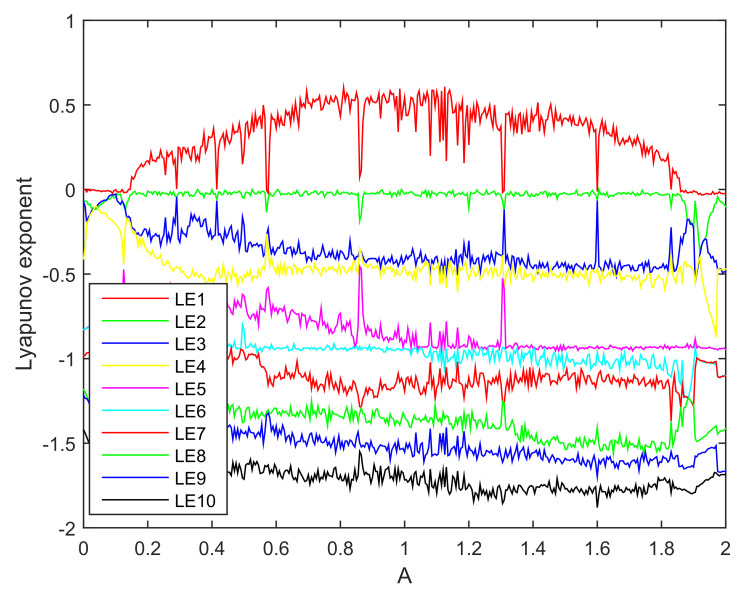
Lyapunov exponent spectrum of a tenth-order chaotic system.

The Lyapunov exponent spectrum is shown in Figure 8.

From Figure 8, For A∈[0.15,1.86], except of small windows in which the LLE is equal to zero, the largest Lyapunov exponents is positive, thereby confirming that the system is chaotic. For A<0.15 and A>1.86 and some aforementioned small windows inside the chaotic region, the LLE is equal to zero, the system in these regions exhibits limit cycle.

## 6. Problems and Future Work

In this work, there are two things which is worth to study in future.

1. The equations for numerical calculation and its corresponding circuit is not exactly the same. We ran many experiments. From the dynamic behavior aspect, the equations and the circuit should belong to one chaotic system because the phase space plot look like the same. However, if the designed circuit is strictly consistent with the corresponding equations, the system cannot exhibit chaos, or vice versa.
2. The Lyapunov exponent will exhibit a mutation in limit cycle range. For example, in Figure 5, when A∈[1.690,1.715], the second Lyapunov exponent (SLE), third Lyapunov exponent (TLE) and fourth Lyapunov exponent (FLE) will not follow the previous trend. The TLE and FLE have a upward movement while the SLE has a downward movement. In this range, the SLE and TLE are equal, while the FLE is equal to the previous value of TLE.

## 7. Conclusions

This paper proposed a simple class of chaotic systems with hyperbolic sine nonlinearity. A novel nth-order ordinary differential equation has been proposed for a generation of various chaotic systems. Any desirable order of hyperbolic sine chaotic systems can be constructed via the proposed method. In this paper, fourth-order, fifth-order, and tenth-order chaotic systems are taken as examples. The dynamic mechanism of these systems has been investigated by analyzing the Lyapunov exponents spectrum. Two back-to-back diodes are used to approximate hyperbolic sine nonlinearity without any multiplier or subcircuits. Thus, the physical circuits are very easy to construct, making it possible for them to achieve both physical simplicity and analytic complexity at the same time.
